# Optimizing Hemp Fiber Production for High Performance Composite Applications

**DOI:** 10.3389/fpls.2018.01702

**Published:** 2018-11-23

**Authors:** Salvatore Musio, Jörg Müssig, Stefano Amaducci

**Affiliations:** ^1^Department of Sustainable Crop Production, Università Cattolica del Sacro Cuore, Piacenza, Italy; ^2^The Biological Materials Group, Biomimetics, Hochschule Bremen, City University of Applied Sciences Bremen, Bremen, Germany; ^3^Gruppo Fibranova srl, Bientina, Italy

**Keywords:** hemp, yellow stem, retting, fiber quality, IFBT, high performance composites

## Abstract

Hemp is a sustainable and environmental friendly crop that can provide valuable raw materials to a large number of industrial applications. Traditionally harvested at full flowering for textile destinations, nowadays hemp is mainly harvested at seed maturity for dual-purpose applications and has a great potential as multipurpose crop. However, the European hemp fiber market is stagnating if compared to the growing market of hemp seeds and phytocannabinoids. To support a sustainable growth of the hemp fiber market, agronomic techniques as well as genotypes and post-harvest processing should be optimized to preserve fiber quality during grain ripening, enabling industrial processing and maintaining, or even increasing, actual fiber applications and improving high-added value applications. In this paper, the effect of genotypes, harvest times, retting methods and processing on the yield and quality of long hemp for wet spun yarns was investigated. Conventional green-stem varieties were compared with yellow-stem ones on two harvesting times: at full flower and seed maturity. Scutching was performed on un-retted stems and dew-retted stems, the un-retted scutched fiber bundles were then bio-degummed before hackling. Both scutching and hackling was performed on flax machines. Quality of hackled hemp, with particular reference to its suitability for high performance composites production, was assessed. The results of fiber extraction indicate that yellow-stem varieties are characterized by higher scutching efficiency than green-stem varieties. Composites strength at breaking point, measured on specimens produced with the Impregnated Fiber Bundle Test, was lower with hemp obtained from stems harvested at seed maturity than at full flowering. On average, back-calculated fiber properties, from hackled hemp-epoxy composites, proved the suitability of long hemp fiber bundles for high performance composites applications, having properties comparable to those of high quality long flax.

Highlights:

- The trait yellow stem in hemp is an indicator of processability.

- Yellow stem varieties have finer hackled fiber bundles.

- Controlled dew retting increased yield of hackled fiber compared to bio-degumming.

- Retting influenced fiber and composite mechanical properties.

- Hemp can achieve properties comparable to high quality long flax for high performance composites.

## Introduction

**FIGURE 1:**
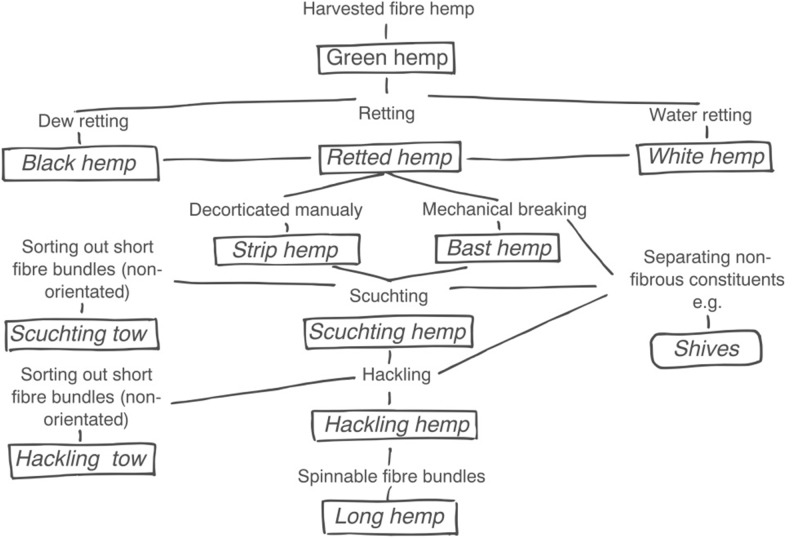
A schematic overview of the systematic nomenclature used in traditional hemp processing (adapted from Schnegelsberg, 1999).

Hemp is a high yielding, sustainable, and environmental friendly crop that can provide valuable raw materials to a large number of applications (Carus et al., 2013). Hemp has been grown for centuries in Europe for the high quality of its fiber that was used to produce ropes, clothing, and paper (Ranalli and Venturi, 2004). Traditionally harvested at full flowering to optimize long fiber bundle extraction, nowadays hemp is mainly harvested at seed maturity for dual-purpose applications (Tang et al., 2016) and has a great potential as multipurpose crop (Amaducci et al., 2015). The economic value of hemp can be maximized if all plant biomass (stems, inflorescences and seeds) is exploited; delaying harvest until the generative phase is completed (Calzolari et al., 2017). For this, agronomic techniques and genotypes should be adapted to preserve fiber quality during grain ripening, enabling industrial processing and maintaining, or even increasing, actual fiber applications (Amaducci et al., 2008c, 2015; Tang et al., 2016, 2017). Hemp processability, or improved decorticability, was a breeding target for yellow stemmed varieties, as Chamaeleon (Toonen et al., 2004; Bennett et al., 2006) and Carmaleonte (Grassi and McPartland, 2017). In Europe, hemp stems are processed mostly in the “disordered line” (Amaducci and Gusovious, 2010) producing not aligned fiber bundles (often not systematically and incorrectly identified as “technical fiber") (Carus et al., 2013) used in the automotive industry (Taylor et al., 2005; Pecenka et al., 2009; Müssig, 2010) and for the production of paper (Angelova et al., 2004; González-García et al., 2010) and bio-based composites (Saleem et al., 2008; Yan et al., 2013). Several authors have investigated the production of long hemp (Figure 1) processed with flax scutching and hackling machines, the so-called “longitudinal line" (Amaducci and Gusovious, 2010), for textile and high-added value applications (Amaducci, 2005a; Turunen and van der Werf, 2006; Amaducci et al., 2008b; Müssig, 2010).

The possibility to obtain long aligned fiber bundles from hemp is limited by the lack of dedicated harvesting machines that can mow hemp stems, lay them on the field in aligned swaths and cut them in 1 m long portions, so that they can be fed in flax scutching lines (Amaducci, 2003; Venturi et al., 2007; Amaducci and Gusovious, 2010). Further bottlenecks, that hamper the production of long hemp suitable for traditional wet spinning, are the retting process and the availability of processing machines suitable for hemp. Retting is a biochemical process, in which enzymes produced by microorganisms attack the pectins that glue together fiber cells, aiding the separation of fiber bundles within the bast fiber and of shives from bast fiber (Booth et al., 2004; Akin, 2010). Microbiological retting can be carried out in water (water retting) or on the soil (dew retting). In the past, the retting process was carried out in open water basins. Nowadays water-retting is considered to have a high environmental impact, due to high water use (Turunen and van der Werf, 2006) and high oxygen demand (BOD) of the waste waters (Keller et al., 2001). The impact of water retting is reduced in the case of controlled warm water retting, where the retting process is optimized with target bacterial inoculum (Di Candilo et al., 2010) and performing retting on un-retted scutched hemp (Amaducci et al., 2008a; Zatta et al., 2012). Dew retting, instead, is carried out on the field, where the stems are left after harvesting, mainly by fungi without the use of water and only relying on rain or air humidity (dew). This process, widespread in Europe for its low economic impact (Jankauskienė et al., 2015), is strictly dependent on microclimatic conditions (Thomsen et al., 2005) and produces un-homogeneous results. The influence of uncontrollable microclimatic conditions on the dew retting process is extremely high: over-retting and under-retting can occur frequently, affecting hemp fiber quality (Müssig and Martens, 2003; Jankauskienė et al., 2015). In order to avoid degradation of cellulose, a mixture of selected enzymes or fungi can be spread on the stems (Liu et al., 2017) resulting in a reduced dew retting duration characterized by a low cellulosic activity. Harvesting technique (Müssig and Martens, 2003), retting method (Placet et al., 2017) as well as agronomic practice (Amaducci et al., 2002a,b, 2005b, 2008a; Westerhuis et al., 2009) and genotype (Sankari, 2000; Amaducci et al., 2002a,b, 2008b; Westerhuis et al., 2009; Jankauskienė et al., 2015; Müssig and Amaducci, 2018) are important determinant of fiber quality, that affect the possibility to use hemp fiber for high-added value application as textile and high performance bio-based composites. In the frame of the Multihemp project a longitudinal long fiber bundle extraction process (Amaducci and Gusovious, 2010) was investigated, where long hackled hemp was used for high performance composites while the losses of this process (hackling tow) were used to investigate the preparation of mid performance bio-based composites.

In this paper, we investigated how hemp fiber quality, with particular reference to its suitability for high performance composites production, is affected by genotype (in particular comparing new yellow stemmed varieties to conventional ones), harvest time and retting method. Impregnated fiber bundle tests (Bensadoun et al., 2017) were carried out to compare composites and back-calculated fiber properties of hackled fiber bundles obtained from contrasting genotypes, harvest times and retting methods, and to compare hemp fiber performance with that of flax (Bensadoun et al., 2017).

## Materials and Methods

### Field Trial, Microclimatic Condition, and Tested Variety

The field trial was carried out in Piacenza, Italy (45°N, 9°E, 60 m a.s.l.) in 2016 with four monoecious varieties (Table 1) planted in large-scale plots (700 m^2^). Three sub-plot of 20 m^2^ were selected, for each variety, at emergence to carry out analysis relative to plant biometry, biomass yield and to collect the stems needed for lab-decortication measurements. Sowing was carried out on 18^th^ of April using an experimental sowing machine (Hege 90, Hege Maschinen GmbH, Waldenburg, Germany) with an inter-row distance of 15 cm. All the plots were sown with a target density of 120 viable seeds m^-2^ and, 35 days after sowing, were fertilized with 60 kg ha^-1^ of nitrogen, as recommended in a previous study (Tang et al., 2017). To prevent water stress, irrigation was carried out twice (at the beginning and the end of July), distributing a total amount of 61 mm of water. Average temperature, between April and October, was 18.6°C. The total amount of rainfall in the same period was 255 mm. This research was carried out in the frame of the Multihemp project that promotes the use of hemp as a multipurpose crop, for the production of fiber and seeds. The target harvesting time in Multihemp was therefore at seed maturity, however, two (Carmaleonte and Futura 75) of the four varieties compared in this study (Table 1) were also harvested at full flowering to assess the influence of harvest time on the suitability of hemp fiber for the production of high performance composites.

**Table 1:** List of the varieties used for the large-scale trial.

Variety commercial name	Breeder	Stem color	Harvest at female full flowering stage (H1)	Harvest at seed maturity stage (H2)
Carmaleonte	Istituto sperimentale per le colture industriali (CREA-CIN), Bologna (IT)	Yellow	17/08/2016	20/09/2016
Fédora 17	Fédération Nationale des Producteurs de semences de Chanvre (FNPC), Le Mans (FR)	Green	/	07/09/2016
Furura 75	Fédération Nationale des Producteurs de semences de Chanvre (FNPC), Le Mans (FR)	Green	19/08/2016	20/09/2016
Ivory	Vandijke Research BV, Scheemda (NL)	Yellow	/	07/09/2016


### Harvesting and Fiber Processing

**FIGURE 2:**
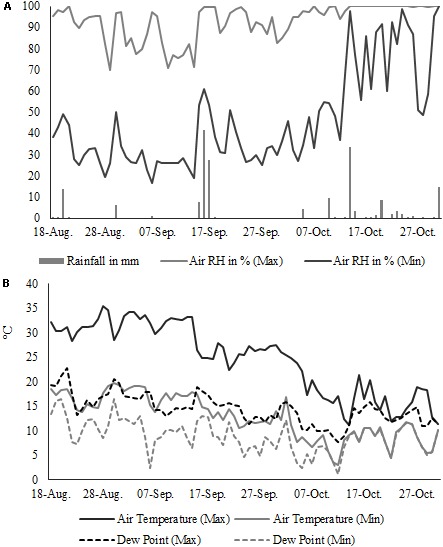
Microclimatic condition during the dew-retting period (daily minimum and maximum): **(A)** air temperature (°C), dew point (°C) and **(B)** air humidity (%) and rainfall (mm).

Harvesting was carried out using a mower (FB-940, Gaspardo Campodarsego, Italy) mounted on a tractor (MF-240, Massey Ferguson, Duluth, GA, United States); the harvested plants were parallelized and cut at 10 and 110 cm starting from the base of the stem. 1-m stem portions were divided into two groups. The first group of stems, which will be referred to as “un-retted stems,” were air-dried under a roof, paying attention that no retting of the stems occurred. The stems were then scutched and the resulting long scutched fiber bundles were bio-degummed. The second group was left in the field in an ordered windrow for dew retting and field drying. Dew retting, that was only carried out on stems of Carmaleonte and Futura 75, lasted 33 and 46 days after the first harvest (H1) and 21 and 35 days after the second harvest (H2), respectively. Retting degree was determined by expert assessment on the basis of stem color and by observing the level of separation of shives from bast fiber during manual breaking of the stems. Adverse weather condition in October (Figure 2) prevented stem drying and as a consequence stems collected at seed maturity (H2), of both varieties, were over retted and were discarded during the scutching process.

Scutching of un-retted and dew-retted stems was performed in collaboration with the companies Linificio e Canapificio Nazionale (Villa d’Almè, Italy) and Térre de Lin (Saint-Pierre-le-Viger, France) with a modern flax scutching line. After scutching, un-retted fibers bundles were warm water retted in the laboratories of the company Gruppo Fibranova (Pisa, Italy), with an innovative bio-degumming process (Amaducci et al., 2008a). This method is based on the creation and maintenance of a microbial retting population. Un-retted fiber bundles were immersed in the retting liquor and bio-degummed for about 90 h keeping the liquor at 30°C. The degree of fiber bundles separation was determined from visual and tactile evaluation by Gruppo Fibranova operator. The bio-degumming system is paired with a monitoring system for temperature, pH and oxidation–reduction potential of the liquor; average, minimum, and maximum for main bio-degumming parameters are summarized in Table 2. After bio-degumming, fiber bundles were washed with tap water to remove retting liquor residues (cleaning). Hackling of dew-retted and bio-degummed fiber bundles was carried out in collaboration with Linificio e Canapificio Nazionale and Lietlinen (Kaunas, Lithuania) in an industrial discontinuous hackling system for flax (Linmack machine of Mackie International).

**Table 2:** Duration and monitored parameters of the bio-degumming system.

	Bio-degumming duration in h	Temperature in °C	pH
Average	93	30.7	8.1
Maximum	118	32.5	8.2
Minimum	69	26.1	8.0


Ten stems, in three replicates per each sample, were processed using a lab-scaled hemp stem decorticator (Worthmann Maschinenbau GmbH, Barßel-Harkebrügge, Germany) following the methodology described in Wang et al. (2018). All samples were conditioned at 20 ± 3°C and a relative humidity of 60 ± 5% for 48 h, before processing. Each stem was passed six times through the decorticator, composed by four pairs of profiled rolls. Sample mass was recorded before breaking (m_0_), after two and six decortication steps (m_2_ and m_6_) and after manual removing of residual shives attached to fiber bundles (m_7_). Recorded data were used to calculate bast fiber content after decortication (BCD), shives content after decortication (χ) and two decortication efficiency indexes relative to the amount of shives, on the total shives content, detached after two (η_dec1_) and six (η_dec2_) passages through the breaker (Wang et al., 2018).

### Fiber Analysis

Fineness of the scutched and hackled fiber bundles was measured using the Fineness Maturity Tester (FMT-Shirley) (Shirley Developments, Ltd., Stockport, England) (Müssig, 2001; Linger et al., 2002). Indirect fineness measurement with the FMT-Shirley device is carried out by examination of the fiber surface by a flow of air. Shirley values are low when measuring coarse objects and high with fine objects. Before measurements, fiber bundles were conditioned for 24 h at 20°C and 65% relative humidity. A random sample of 4 g was weighted and tested twice with the FMT-Shirley at low-pressure (1 l min^-1^) compression stage (PL). For each sample, three specimens were tested. A subsample of hackled hemp fiber bundles, was parallelized and cut for scanning electron microscope (SEM) preparation according to Slootmaker and Müssig (2010). Examinations were carried out using a JSM-6510 SEM (JEOL GmbH, Eching, Germany) at an acceleration voltage of 20 kV. All specimens were mounted on aluminum holders using double-sided electrically conducting carbon adhesive and sputtered for 1 min, with approximately 50 mA, with a layer of gold prior to SEM observations using a Bal-Tec sputter coater type SCD 005 (Bal-Tec AG, Balzers, Liechtenstein).

### High Performance Composites Preparation and Test

The “impregnated fiber bundle test” (IBFT) (Bensadoun et al., 2017) was carried out using hackling hemp (Figure 1) samples from the varieties Carmaleonte and Futura 75. The IBFT was performed according to ISO 10618:2004 standard for carbon fiber following the modification proposed by Bensadoun et al. (2017) for long flax. The hackled fiber bundles were cut into a length of 25 cm in the middle part of the hackled fiber bundles. The samples were pre-dried at 60°C for 18 h and divided into six subsamples, each weighing 3.7 g. This value was calculated using a hemp fiber density of 1.48 g cm^-3^ (Li and Pickering, 2009) and a target fiber volume fraction of 50%. The fiber samples were placed in the mold cavity, where a vacuum film was previously laid to create a unidirectional composite (Bensadoun et al., 2017). The resin, 50 g of injection resin EPIKOTE^®^ RIMR 135 and 15 g of hardener EPIKURE^®^ RIMH 137 (Lange+Ritter GmbH, Gerlingen, Germany), was poured on the top of the fibers. The vacuum film was then folded and the counter-mold was placed on the top and fixed using spacers for determining the sub-samples’ thickness. After 48 h of curing at room temperature, the sub-samples were demolded and post-cured in the heating oven (Memmert, Schwabach, Germany) at a temperature of 60°C for 18 h. Mass and dimensions of the rectangularly shaped composites were recorded to calculate the real fiber volume fraction. The measured fiber volume fraction (29.6 ± 1.6%) was normalized to 30%, after biometric measurements, using the rule of mixture (Gibson, 2016). The sub-samples length was reduced to 20 cm, cutting away the extremities, and a glass fiber reinforcement, 2 mm thick, was glued on each extremity to avoid slippage and to distribute the force applied on the sample by the clamps. These were cured for 24 h at room temperature followed by 14 h at 50°C. Tensile tests were performed along the longitudinal direction of the sub-sample after a conditioning period of 24 h at 23 ± 1°C and 50% RH ± 2%. Tensile properties of the composites were tested with a Zwick/Roell universal testing machine (Z020) equipped with a 20 kN load cell (Zwick/Roell, Ulm, Germany) with a displacement speed of 2 mm/min. The gauge length was 150 mm. Tests were followed by video extensometer (VideoXtens, Zwick, Ulm, Germany). The stiffness was calculated between 0 and 0.1% strain (E_1_) and 0.3 and 0.5% strain (E_2_) as reported by Bensadoun et al. (2017) for flax-composites tests. A SEM investigation was used to study the fracture surface of the composite samples.

### Statistical Analysis

Data recorded with the lab-scaled stem decorticator were analyzed with a one-way ANOVA, with a confidential interval of 95%. Because of the unbalanced dataset design (two varieties on four were harvested two times and only plants harvested at full flower were dew retted), comparison of mean between the eight groups was carried out. Differences between each group were evaluated using the Tukey multiple comparison test. Bast fiber content, fineness and composites properties, measured on Carmaleonte and Futura 75, were analyzed with a two-way ANOVA with a confidential interval of 95%. The *post hoc* single-step multiple comparisons were done with Tukey method if the effect of one factor on a dependent variable was significant. Two separated analysis were carried out. (i) Harvest time effect was investigated on un-retted scutched fiber bundles that were subsequently bio-degummed, while (ii) retting treatment effect was assessed on plant material harvested at full flower (H1). The calculations were performed using the software R (R Core Team, 2012).

## Results

**FIGURE 3:**
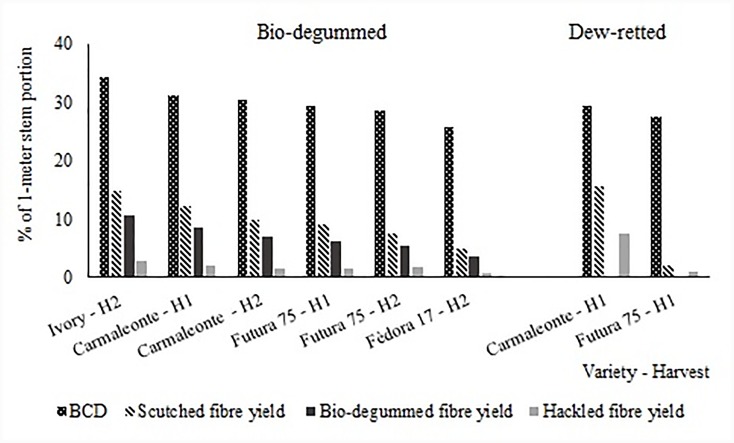
Yields of the different fiber extraction step in percentage of the 1-m hemp stem portion: bast fiber content (BCD), scutched fiber yield, bio-degummed fiber yield, and hackled fiber yield.

Average plant height was in all cases above 2 m. The shortest variety at seed maturity was Fédora 17, while Futura 75 was the tallest one (2310 and 3100 mm respectively). Stem length increased between harvest times, by 18.0 and 16.7% in Carmaleonte and Futura 75 respectively. Stem diameters ranged between 7 and 10 mm, the thinnest stems were found in Carmaleonte at full flowering and in Fédora 17 at seed maturity. Futura 75 had the thickest stems at seed maturity (10.3 mm). Stem biomass yield was highest in the 1^st^ harvest, at full flowering (H1), for both varieties harvested at this stage (Carmaleonte and Futura 75, 13.5 and 12.5 Mg ha^-1^ respectively). At seed maturity, when the second harvest was carried out, total biomass ranged from 5.8 to 11.2 Mg ha^-1^ while stem biomass ranged 3.9 to 8.4 Mg ha^-1^ for Futura 75 and Ivory respectively. A decrease of plant density from the first to the second harvest time was observed in Carmaleonte and Futura 75 as a consequence of a severe attack of Fusarium wilt, which caused the premature senescence of the affected plants. The disease spread during the phase of seed ripening and the senescent plants remained in a standing position. Plants affected by wilt were discarded after harvesting and quality measurements were carried out on healthy stems only. Bast fiber content (%), separated from the shives with the lab-scaled hemp stem decorticator, was significantly different between groups (*p* < 0.001). The highest bast fiber content after decortication (BCD) was recorded in Ivory and the lowest in Fédora 17, both harvested only at H2 (Table 3, dotted bars in Figure 3). Initial decortication efficiency (η_dec1_), namely the percentage of removed shives on total shives content after two decortication steps, ranged between 47.4 and 79.1% (Table 3). η_dec1_ was lower in Fédora 17 than in Ivory, Carmaleonte (H2) and Futura 75 (H1) (*p* < 0.01). Total decortication efficiency (η_dec2_), or the highest efficiency of mechanical decortication, ranged between 80.1 and 95.9%. The lowest η_dec2_ was registered in Fédora 17 (*p* < 0.001). Shives content after decortication (χ) ranged between 9.1 and 32.8% (*p* < 0.001). Despite total decortication efficiency not being significantly different between retting treatments in Futura 75 (H1), shives percentage on decorticated fiber was lowest in dew retted hemp (Table 3).

**Table 3:** Bast fiber content (BCD in %), initial decortication efficiency (η_dec1_ in %), total decortication efficiency (η_dec2_ in %) and shives content after decortication (χ in %).

Variety	Harvest	Retting treatment	BCD	η_dec1_	η_dec2_	X
Carmaleonte	H1	Un-retted	31.1 ± 3.4 b	59.4 ± 23.7 ab	89.2 ± 10.8 bc	17.0 ± 14.4 bc
Carmaleonte	H1	Dew-retted	29.5 ± 3.6 bc	61.2 ± 22.0 ab	91.6 ± 7.9 bc	15.3 ± 12.2 bc
Carmaleonte	H2	Un-retted	30.4 ± 3.3 b	63.0 ± 25.6 b	91.8 ± 8.8 bc	14.3 ± 12.8 bc
Fédora 17	H2	Un-retted	25.8 ± 3.5 d	47.4 ± 23.2 a	80.1 ± 14.5 a	32.8 ± 19.1 a
Futura 75	H1	Un-retted	29.3 ± 4.1 bc	63.9 ± 18.9 b	89.0 ± 8.4 bc	19.6 ± 12.3 b
Futura 75	H1	Dew-retted	27.6 ± 3.1 cd	79.1 ± 11.7 b	95.9 ± 4.1 c	9.1 ± 8.1 c
Futura 75	H2	Un-retted	28.6 ± 4.0 bc	60.6 ± 10.7 ab	86.6 ± 9.2 b	23.1 ± 13.1 ab
Ivory	H2	Un-retted	35.5 ± 3.3 a	65.2 ± 19.7 b	91.2 ± 6.2 bc	13.4 ± 9.5 bc


*Values within a column followed by the same letter are not significantly different at the *p* = 0.05 level by Tukey *post hoc* test.*

The yield of scutching hemp (hatched bars in Figure 3) on un-retted stems ranged between 3.5 and 14.7%, while it ranged from 2.1 to 15.6% in dew retted stems. The yield of scutched fiber bundles was higher for dew retted specimens of Carmaleonte compared to bio-degummed specimens, while it was the other way round for Futura 75.

Long scutched hemp obtained from un-retted stems was bio-degummed (dark gray bars in Figure 3). No differences were observed among varieties and between harvest times regarding the relative bio-degumming losses. On average, the loss of primary fiber mass and secondary fiber during bio-degumming was 27.8 ± 1.6%.

In order to refine and parallelise fiber bundles and make them suitable for high-value applications (bio-composites and textiles) all bio-degummed and dew retted fiber samples were hackled. The range of hackling yield was quite large with the highest value obtained for Carmaleonte H1 dew-retted and the lowest for Fédora 17 H2. Hackling yield (percentage of hackled hemp over feeding scutched retted hemp into the hackling device) was not influenced by variety and harvest time but only by retting treatment. On average bio-degummed hemp had a much lower hackling yield (25.0 ± 3.7%) than dew-retted hemp (48.5 ± 2.0%).

Composite properties are directly dependent on the mechanical properties of their constituents (fibers and matrix). The quality key figures of cellulosic fibers for high-added values applications are low density, fineness, high specific tensile strength, and stiffness (Placet, 2009). Considering the statistical analysis carried out on samples from Carmaleonte and Futura 75 (for which all the combinations of treatments were available), the effect of genotype influenced most of the fiber quality traits analyzed in our research; only fineness of scutched fiber bundles (F_s_), composites strength (σ) and back-calculated fiber strength (σ_f_) were not significantly different between the two varieties (Table 4). The effect of harvest time was low and it was significant only for the following parameters: fineness of the scutched fiber bundles (F_s_), strength (σ, σ_f_) and stiffness (E_1_, E_1f_ and E_2_, E_2f_). The interaction variety × harvest time was significant only for the fineness of scutched fiber bundles (F_s_) and stiffness (E_2_, E_2f_). The retting treatment significantly affected BCD, fineness of the scutched fiber bundles (F_s_) and stiffness (E_2_, E_2f_). Variety × retting treatment interaction significantly affected fineness of the scutched fiber bundles and stiffness (E_1_, E_1f_ and E_2_, E_2f_).

**Table 4:** Results of the two-way ANOVA used to investigate the effect of variety, harvest time, retting treatment and the interactions “variety × harvest” and “variety × retting treatment” on: bast fiber content (BCD), fineness of the scutched fiber bundles (F_s_), fineness of the hackled fiber bundles (F_h_), composites strength (σ), composites Young’s modulus in the interval of elongation 0–0.1% (E_1_), composites Young’s modulus in the interval of elongation 0.3–0.5% (E_2_), back calculated fiber strength (σ_f_), back calculated fiber Young’s modulus in the interval of elongation 0–0.1% (E_1f_) and back calculated fiber Young’s modulus in the interval of elongation 0.3–0.5% (E_2f_).

	Variety	Harvest	VxH	Retting treatment	VxR
BCD	**6.93–0.01 ^∗∗^**	1.05–0.31 ns	0.0–0.98 1.0 ns	**6.94**–**0.01 ^∗∗^**	0.07–0.79 ns
F_s_	0.84–0.37 ns	4.06–0.06 ns	**12.11**–**0.00 ^∗∗^**	**11.10**–**0.00 ^∗∗^**	**4.93**–**0.04 ^∗^**
F_h_	**24.32**–**8e^-05^^∗∗∗^**	2.46–0.13 ns	1.26–0.28 ns	0.62–0.44 ns	2.20–0.15 ns
σ	0.43–0.52 ns	**6.95**–**0.02 ^∗^**	0.19–0.67 ns	0.48–0.50 ns	1.13–0.30 ns
E_1_	**8.98**–**0.01 ^∗∗^**	3.35–0.08 ns	1.17–0.29 ns	0.40–0.54 ns	**5.56**–**0.03 ^∗^**
E_2_	**31.71**–**1.6e^-05^^∗∗∗^**	**5.44**–**0.03 ^∗^**	3.30–0.08 ns	3.69–0.07 ns	**7.66**–**0.01 ^∗^**
σ_f_	0.43–0.52 ns	**6.95**–**0.02 ^∗^**	0.19–0.67 ns	0.48 –0.50 ns	1.13 –0.30 ns
E_1f_	**8.98**–**0.01 ^∗∗^**	3.35–0.08 ns	1.17–0.29 ns	0.40–0.54 ns	**5.56**–**0.03 ^∗^**
E_2f_	**31.71**–**1.6e^-05^^∗∗∗^**	**5.44**–**0.03 ^∗^**	3.30–0.08 ns	3.69–0.07 ns	**7.66–0.01 ^∗^**


*The significance values are indicated in bold. ^∗^*p* < 0.05; ^∗∗^*p* < 0.01; ^∗∗∗^*p* < 0.001.*

Bast fiber content after decortication for Carmaleonte and Futura 75 harvested at full flowering was affected by variety and retting treatment (Table 4). Carmaleonte had the highest BCD values: 31.1 ± 3.4% in un-retted stems and 29.5 ± 3.6% in dew retted stems. The effect of the retting treatment on BCD, described for Carmaleonte, was found also in Futura 75, with un-retted stems having BCD 6.5% higher than dew retted stems (29.3 ± 4.1 and 27.4 ± 3.4%, respectively). BCD was not significantly affected by harvest time.

The fineness of scutched and hackled fiber bundles, measured with the FMT-Shirley confirmed that scutched fiber bundles were coarser than hackled ones. Finesses of scutched fiber bundles increased significantly (higher PL values) in the second harvest only in Carmaleonte while no significant differences among harvest times were observed in Futura 75 (VxH, *p* < 0,05, Table 4). Dew-retted scutched fiber bundles were on average finer than un-retted scutched fiber bundles, especially in Carmaleonte. The fineness of hackled fibers bundles was significantly affected by variety, with the highest values found in Carmaleonte, in particular in dew retted fiber bundles, but it was not influenced by retting treatment and harvest time (Table 5).

**Table 5:** Results of the statistical analysis on PL values of scutched fiber bundles (F_s_) and hackled fiber bundles (F_h_): “variety × retting treatment” (Bio-degummed and Dew retted) and “variety × harvest time” (H1: full flowering stage, H2: seed maturity stage).

Variety	Retting treatment	F_s_	F_h_
Carmaleonte	Bio-degummed	5.1 ± 0.6 b	10.6 ± 0.8 a
	Dew-retted	6.3 ± 0.4 a	12.1 ± 1.4 a
Futura 75	Bio-degummed	5.7 ± 0.5 ab	9.2 ± 1.0 b
	Dew-retted	5.9 ± 0.7 ab	9.0 ± 1.5 b
Average	Bio-degummed	5.4 ± 0.6 b	9.9 ± 1.1 ns
	Dew-retted	6.1 ± 0.6 a	9.0 ± 2.1 ns

**Variety**	**Harvest time**	**F_s_**	**F_h_**

Carmaleonte	H1	5.1 ± 0.6 b	10.6 ± 0.8 a
	H2	6.3 ± 0.5 a	10.4 ± 1.1 a
Futura 75	H1	5.7 ± 0.5 ab	9.2 ± 1.0 b
	H2	5.3 ± 0.6 b	8.2 ± 0.8 b
Average	H1	5.0 ± 0.6 ns	9.1 ± 1.1 ns
	H2	5.5 ± 0.6 ns	9.0 ± 1.2 ns


*Values within a column followed by the same letter are not significantly different at the *p* = 0.05 level by Tukey post hoc test.*

**FIGURE 4:**
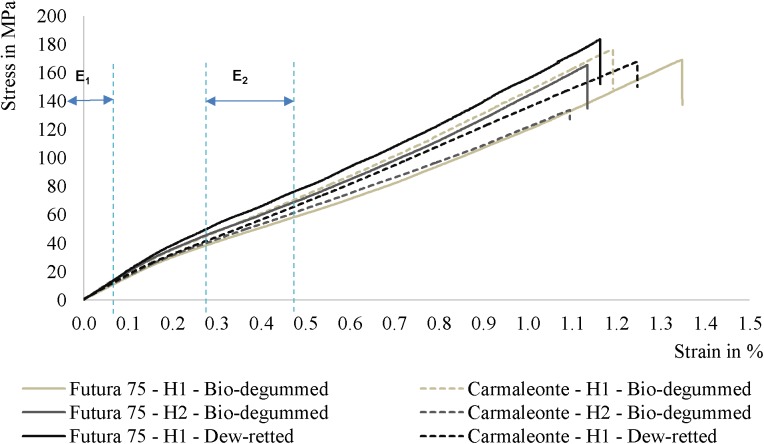
Stress-strain curves of the composite made by hackled hemp fiber bundles from the varieties Carmaleonte and Futura 75 (H1: harvest at full flowering stage; H2: harvest at seed maturity stage).

The stress-strain curves of three composite samples, one for each combination of treatments [bio-degummed, harvested at full flowering (1) and at seed maturity (2) as well as dew retted, harvested at full flowering stage (3)] are presented for Carmaleonte and for Futura 75 (Figure 4). The stress intervals used for stiffness calculation (slope of the stress-strain curve) are highlighted, too (E_1_, E_2_).

The analysis of the strength (σ) at breaking point shows a significant effect of harvest time (Table 6) with an average value of 155 and 146 MPa in the first and in the second harvest, respectively. No significant differences were detected between varieties and retting treatments. Both stiffness values (E_1_ and E_2_) were significantly different between varieties. Carmaleonte had the highest stiffness in both of the strain intervals observed (E_1_ and E_2_). Harvest time significantly affected stiffness measurements in E_2_, it was higher in the 1^st^ harvest than in the 2^nd^ one. Stiffness was not affected by retting treatment; however, E_1_ and E_2_ in Futura 75 bio-degummed samples were significantly lower than in dew-retted ones (“variety × retting treatment,” *p* < 0.05). The same trends described for bio-composites properties were found in the back-calculated fiber properties (Table 7).

**Table 6:** Results of the statistical analysis on bio-composites properties: strength at breaking point (σ) in MPa and modulus (E_1_ and E_2_) in GPa: “variety × retting treatment” and “variety × harvest time” (H1: full flowering stage; H2: seed maturity stage).

Variety	Retting treatment	σ	E_1_	E_2_
Carmaleonte	Bio-degummed	163 ± 19.7 ns	19.5 ± 1.3 a	12.6 ± 1.0 a
	Dew-retted	176 ± 7.9 ns	18.2 ± 1.4 a	12.3 ± 1.0 a
Futura 75	Bio-degummed	173 ± 25.2 ns	16.8 ± 1.6 b	10.2 ± 0.7 b
	Dew-retted	170 ± 12.3 ns	19.2 ± 2.9 a	11.9 ± 0.9 a
Average	Bio-degummed	168 ± 22.1 ns	18.2 ± 2.0 ns	11.4 ± 1.5 ns
	Dew-retted	173 ± 10.3 ns	18.7 ± 2.2 ns	12.1 ± 0.9 ns

**Variety**	**Harvest time**	**σ**	**E_1_**	**E_2_**

Carmaleonte	H1	163 ± 19.7 ns	19.5 ± 1.3 a	12.6 ± 1.0 a
	H2	144 ± 8.4 ns	17.6 ± 1.3 a	11.2 ± 0.4 a
Futura 75	H1	173 ± 25.2 ns	16.8 ± 1.6 b	10.2 ± 0.7 b
	H2	146 ± 27.2 ns	16.3 ± 2.1 b	10.9 ± 0.9 b
Average	H1	155 ± 22.1 a	16.8 ± 2.0 ns	10.5 ± 1.5 a
	H2	146 ± 23.4 ns	15.9 ± 1.5 ns	9.9 ± 0.8 b


*Values within a column followed by the same letter are not significantly different at the *p* = 0.05 level by Tukey post hoc test.*

**Table 7:** Results of the statistical analysis on back-calculated fiber bundle properties: strength at breaking point (σ_f_) in MPa and modulus (E_1f_ and E_2f_) in GPa: “variety × retting treatment” and “variety × harvest time” (H1: full flowering stage; H2: seed maturity stage).

Variety	Retting treatment	σ_f_	E_1f_	E_2f_
Carmaleonte	Bio-degummed	420 ± 65.5 ns	58.1 ± 4.4 a	34.9 ± 3.4 a
	Dew-retted	462 ± 26.3 ns	53.5 ± 4.7 a	33.9 ± 3.2 a
Futura 75	Bio-degummed	452 ± 84.0 ns	49.0 ± 5.4 b	26.9 ± 2.3 b
	Dew-retted	443 ± 41.1 ns	56.8 ± 9.7 a	32.6 ± 3.0 a
Average	Bio-degummed	436 ± 73.7 ns	53.5 ± 6.7 ns	30.9 ± 5.0 ns
	Dew-retted	453 ± 34.4 ns	55.2 ± 7.5 ns	33.2 ± 3.0 ns

**Variety**	**Harvest time**	**σ_f_**	**E_1f_**	**E_2f_**

Carmaleonte	H1	420 ± 65.5 ns	58.1 ± 4.4 a	34.9 ± 3.4 a
	H2	356 ± 27.8 ns	51.6 ± 4.4 a	30.4 ± 1.2 a
Futura 75	H1	452 ± 84.0 ns	49.0 ± 5.4 b	26.9 ± 2.3 b
	H2	362 ± 90.6 ns	47.4 ± 7.0 b	26.3 ± 3.1 b
Average	H1	402 ± 73.7 a	49.4 ± 6.7 ns	28.5 ± 5.0 a
	H2	373 ± 77.9 b	46.4 ± 4.9 ns	26.4 ± 2.5 b


*Values within a column followed by the same letter are not significantly different at the *p* = 0.05 level by Tukey post hoc test.*

**FIGURE 5:**
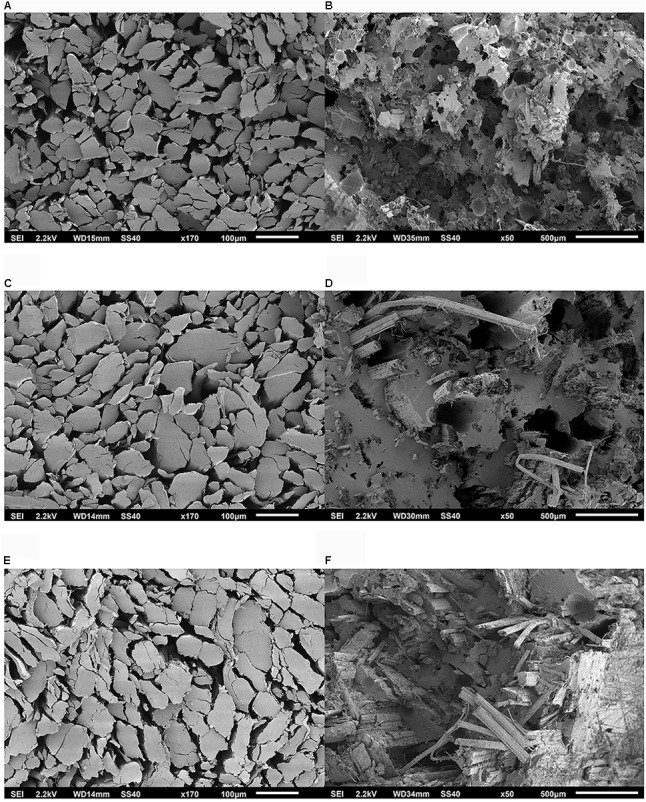
Scanning electron microscope (SEM) micrograph of the samples of the variety Carmaleonte: **(A)** H1 – bio-degummed, fiber bundle transverse section; **(B)** H1 – bio-degummed, composites fraction surface; **(C)** H2- bio-degummed, fiber bundle transverse section; **(D)** H2 – bio-degummed, composites fraction surface; **(E)** H1 – dew-retted, fiber bundle transverse section; **(F)** H1 – dew-retted, composites fracture surface.

**FIGURE 6:**
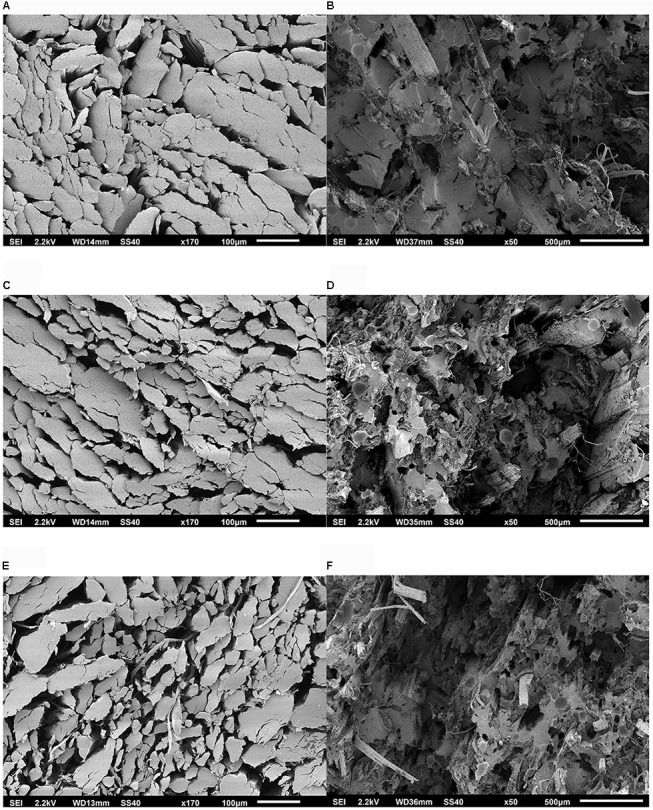
SEM micrograph of the samples of the variety Futura 75: **(A)** H1 – bio-degummed, fiber bundle transverse section; **(B)** H1 – bio-degummed, composites fraction surface; **(C)** H2 – bio-degummed, fiber bundle transverse section; **(D)** H2 – bio-degummed, composites fraction surface; **(E)** H1 – dew-retted, fiber bundle transverse section; **(F)** H1 – dew-retted, composites fraction surface.

The comparison between the two sets of SEM pictures of hackled fiber bundles from Carmaleonte (Figure 5) and Futura 75 (Figure 6) confirmed the results of the FMT-Shirley analysis, which indicated that the finest fiber bundles are found in Carmaleonte. The analysis of composites fracture surface highlights the presence of air bubbles entrapped in the matrix, recognizable as dark-gray light edge spots; while the dark-black spots are the holes left by fiber bundles detached from the matrix.

## Discussion

To promote the use of hemp as a multipurpose crop, harvesting must be carried out at seed maturity, when the production of seeds can be coupled to that of fiber and shives (Amaducci et al., 2008b; Tang et al., 2016, 2017). Delaying stem harvesting until seed maturity poses issues related to stem processability and fiber quality (Keller et al., 2001; Amaducci et al., 2008a). Hemp fiber, obtained at seed maturity, is generally destined to lower value applications such as paper and pulp production (Berbert et al., 2001; Prade et al., 2012; Carus et al., 2013) or in fiber reinforced thermoplastic composites (Wötzel et al., 1999; Shahzad, 2011) using needle felts or fleeces as technical textiles (Carus et al., 2013). To sustain high-value fiber applications from multipurpose hemp, genotypes that can maintain superior fiber quality throughout the seed ripening phase are needed. In this study, yellow genotypes, that were bred to have improved processability and superior fiber traits (van den Broeck et al., 2008; Chandra et al., 2017) were compared to conventional commercial varieties. Comparison between yellow and conventional genotypes was carried out at lab scale, using a lab decorticator (Wang et al., 2018) and on an industrial scutching/hackling line that operates on flax.

The analysis carried out with the lab-scaled hemp stem decorticator indicates that yellow varieties had higher bast fiber content than the conventional ones, that in this study are represented by Futura 75 and Fédora 17 (Figure 3). BCD was higher in Carmaleonte than in Futura 75 (Table 4), while no differences were found between harvest times. In Fédora 17 bast fiber content and decortication efficiency were the lowest (Table 3); these results from lab-scaled decortication validate low scutching yield observed for this variety at industrial scale (Figure 3). On the contrary, the highest decortication efficiency of dew retted stems from Futura 75 is in contrast with the lowest scutching yield at industrial scale. During decortication, the rolls impress force on the shives causing their breaking, while the work of the scutching turbine system affects both shives and bast fiber. Industrial scutching of un-retted stems from two yellow varieties (Carmaleonte and Ivory) and two conventional varieties (Futura 75 and Fédora 17), confirmed that the trait *yellow stem* is an efficient indicator of stem processability. In particular, the yield of scutched fiber bundles was higher in yellow varieties than in conventional ones. These results are in line with those reported by Toonen et al. (2004). The decrease of long scutched hemp yield observed between harvests in Carmaleonte and Futura 75 is not in line with the results of BCD analysis. The decrease in scutched fiber yield from the first to the second harvest time could be related to the development of secondary fibers resulting in increased mechanical resistance of the stems (Liu et al., 2015).

The comparison of scutching yield between un-retted and dew-retted stems confirms that after retting, stems are easier to decorticate (Munder et al., 2004). This is a consequence of the reduction of non-cellulosic components that bond bast fibers to shives and bundles of primary fiber, which in turn decreases the resistance to mechanical processing (Tamburini et al., 2003). However, adverse weather conditions during dew retting can result in over-retting: stems are left too long on the field and cellulolytic enzymes secreted by the microbiota damage fibers reducing long hemp yield (Ribeiro et al., 2015). In this study, for Futura 75, high air humidity, high dew point temperature and low air temperature (Figure 2) favored microbial growth and hampered drying of the stems as a consequence scutching yield was extremely low (2%).

The percentage of hackled fiber bundles obtained from retted samples was similar among varieties, while differences were found between retting treatments. The lower hackling yield obtained with bio-degumming than with dew retting was probably a consequence of the entanglement of fiber bundles that happened during the bio-degumming phase and particularly during the final washing step. The entanglements formed in the fiber bundles reduced the efficiency of the hacking process and decreased significantly hackling yield.

Generally, our investigation confirms the suitability of flax fiber extraction lines for the extraction of long hemp for high-added value applications (Amaducci, 2005a; Turunen and van der Werf, 2006; Amaducci et al., 2008b). In the presented work the scutching and hackling machines were not adjusted for hemp stem; in particular, the scutching line was programmed on a strong set up for flax stem to minimize the presence of residual shives on fiber bundles. A suitable adjustment of the processing machines for hemp stems may improve long hemp yield and quality since hemp stems are thicker compared with flax stems and are subjected to high mechanical stress.

For the production of high-quality fiber for industrial applications, fineness is another desirable trait (Müssig and Martens, 2003; Müssig and Amaducci, 2018). Hackled fiber bundles, as expected, are finer than scutched ones (Table 5). The main purpose of scutching is to remove the shives from the bast fiber bundles, while the refining of bast fiber bundles is carried out during hackling (Bos, 2004; Westerhuis et al., 2009). Fineness of scutched fiber bundles was affected by the retting treatment; in general, dew retted scutched fiber bundles were finer than un-retted one because of the separation among bast fiber bundles provided by the digestion of the middle lamella (Abot et al., 2013). Harvest time affected fineness of scutched specimens only in Carmaleonte (VxH, *p* < 0.01) with finer fiber bundles in the second harvest. The fineness of the hackled fiber bundles was affected by genotype, while no significant differences were observed between harvest times and retting treatments. Fiber bundles from the yellow variety Carmaleonte had higher Shirley values than from Futura 75.

The IFBT for strength at breaking point of the hemp epoxy composites shows a significant effect of harvest time (Table 6) with a decrease of strength at breaking point in the second harvest, in agreement with what was reported by Liu et al. (2015). However, strength at breaking point is more variable at full flowering than at seed maturity in both varieties. Composites and back-calculated fiber properties found in our study were inferior to those obtained in flax using the IFBT method in five different laboratories, as reported by Bensadoun et al. (2017). However, our results are in line with the back-calculated tensile strength of 464 MPa and back-calculated stiffness of 60.5 GPa in the same laboratory and using the same technique for hackled flax fiber bundles (Bensadoun et al., 2017). Moreover, the analysis of the SEM pictures of the hemp-composites fracture surface suggests the possibility to improve composites preparation reducing the bubbles in the matrix and increasing fiber-matrix bounding (Shahzad, 2011). Air-filled cavities in the matrix of the composite (Madsen et al., 2009) can be removed with an under vacuum system, vacuum infusion and compression molding as described by Liu et al. (2016). Interfacial bonding between fiber and matrix can be improved removing non-cellulosic components and increasing fiber bundles separation (Li et al., 2009).

## Conclusion

In this study, a longitudinal hemp line for textile and high-added value applications was investigated. Results obtained at industrial level confirm that yellow stem varieties have higher fiber extraction efficiency than conventional ones. The fineness of hackled fiber bundles was affected by genotype, and was highest in the yellow variety Carmaleonte, but it was not affected by the retting treatment and harvest time. The IFBT technique was used to determine hackled fiber bundle properties by back-calculation from hemp-epoxy composites. Strength and stiffness were higher at full flowering than at seed maturity in both analyzed varieties. The results on composites and back-calculated fiber properties are comparable with those obtained from other authors with long hackled flax and hemp fiber bundles. In this study long hemp fiber bundles, having properties comparable to those of flax, proved to be suitable for high performance composites applications. In particular, these results were underscored for yellow varieties that had the highest decortication efficiency.

## Author Contributions

SM carried out the experiments and wrote the manuscript with support from SA and JM. SA and JM supervised the project. All the authors have read and approved the final manuscript.

## Conflict of Interest Statement

The authors declare that the research was conducted in the absence of any commercial or financial relationships that could be construed as a potential conflict of interest.The handling Editor is currently co-organizing a Research Topic with two of the authors, JM and SA, and confirms the absence of any other collaboration.

## References

[B1] Abot A. Bonnafous C. Touchard F. Thibault F. Chocinski-Arnault L. Lemoine R. (2013). Effects of cultural conditions on the hemp (*Cannabis sativa*) phloem fibres: biological development and mechanical properties. *J. Compos. Mater.* 47 1067–1077. 10.1177/0021998313477669

[B2] Akin D. E. (2010). Flax—structure, chemistry, retting, and processing. *Ind. Appl. Nat. Fibres* 15 89–108. 10.5402/2013/186534 25969769 PMC4403609

[B3] Amaducci S. (2003). HEMP-SYS: design, development and up-scaling of a sustainable production system for hemp textiles – an integrated quality system approach. *J. Ind. Hemp.* 8 79–83. 10.1300/J237v08n02_06

[B4] Amaducci S. (2005a). Hemp production in Italy. *J. Ind. Hemp* 10 109–115. 10.1300/J237v10n01_09

[B5] Amaducci S. Colauzzi M. Bellocchi G. Venturi G. (2008a). Modeling post-emergent hemp phenology (*Cannabis sativa* L.): theory and evaluation. *Eur. J. Agron.* 28 90–102. 10.1016/j.eja.2007.05.006

[B6] Amaducci S. Errani M. Venturi G. (2002a). Plant population effects on fibre hemp morphology and production. *J. Ind. Hemp* 7 33–60.

[B7] Amaducci S. Errani M. Venturi G. (2002b). Response of hemp to plant population and nitrogen fertilisation. *Ital. J. Agron.* 6 103–112.

[B8] Amaducci S. Gusovious H. J. (2010). “‘Hemp– cultivation, extraction and processing,” in *Industrial Applications of Natural Fibres: Structure, Properties and Technical Applications*, ed. Müssig J. (Chichester: John Wiley & Sons, Ltd), 10.1002/9780470660324.ch5

[B9] Amaducci S. Pelatti F. Medeghini Bonatti P. (2005b). Fibre development in hemp (*Cannabis sativa* L.) as affected by agrotechnique: preliminary results of a microscopic study. *J. Ind. Hemp* 10 31–48.

[B10] Amaducci S. Scordia D. Liu F. H. Zhang Q. Guo H. Testa G. (2015). Key cultivation techniques for hemp in Europe and China. *Ind. Crops Prod.* 68 2–16. 10.1016/j.indcrop.2014.06.041

[B11] Amaducci S. Zatta A. Pelatti F. Venturi G. (2008b). Influence of agronomic factors on yield and quality of hemp (*Cannabis sativa* L.) fibre and implication for an innovative production system. *Field Crops Res.* 107 161–169. 10.1016/j.fcr.2008.02.002

[B12] Amaducci S. Zatta A. Raffanini M. Venturi G. (2008c). Characterisation of hemp (*Cannabis sativa* L.) roots under different growing conditions. *Plant Soil* 313 227–235. 10.1007/s11104-008-9695-0

[B13] Angelova V. Ivanova R. Delibaltova V. Ivanov K. (2004). Bio-accumulation and distribution of heavy metals in fibre crops (flax, cotton and hemp). *Ind. Crops Prod.* 19 197–205. 10.1016/j.indcrop.2003.10.001

[B14] Bennett S. J. Snell R. Wright D. (2006). Effect of variety, seed rate and time of cutting on fibre yield of dew-retted hemp. *Ind. Crops Prod.* 24 79–86. 10.1016/j.indcrop.2006.03.007

[B15] Bensadoun F. Verpoest I. Baets J. Müssig J. Graupner N. Davies P. (2017). Impregnated fibre bundle test for natural fibres used in composites. *J. Reinforced Plast. Compos.* 36 942–957. 10.1177/0731684417695461

[B16] Berbert P. A. Queiroz D. M. Sousa E. F. Molina M. B. Melo E. C. Faroni L. R. D. (2001). PH—postharvest technology. *J. Agric. Eng. Res.* 80 65–80. 10.1006/jaer.2000.0689

[B17] Booth I. Goodman A. M. Grishanov S. A. Harwood R. J. (2004). A mechanical investigation of the retting process in dew-retted hemp (*Cannabis sativa*). *Ann. Appl. Biol.* 145 51–58. 10.1111/j.1744-7348.2004.tb00358.x

[B18] Bos H. L. (2004). *The Potential of Flax Fibres as Reinforcement for Composite Materials.* Eindhoven: Technische Universiteit Eindhoven, 10.6100/IR575360

[B19] Calzolari D. Magagnini G. Lucini L. Grassi G. Appendino G. Amaducci S. (2017). High added-value compounds from Cannabis threshing residues. *Ind. Crops Prod.* 198 558–563. 10.1016/j.indcrop.2017.06.063

[B20] Carus M. Karst S. Kauffmann A. (2013). *The European Hemp Industry: Cultivation, Processing and Applications for Fibres, Shivs and Seeds.* Cologne: EIHA, 1–9.

[B21] Chandra S. Lata H. ElSohly M. A. (eds) (2017). *Cannabis sativa L.-Botany and Biotechnology.* Berlin: Springer International Publishing, 10.1007/978-3-319-54564-6

[B22] Di Candilo M. Bonatti P. M. Guidetti C. Focher B. Grippo C. Tamburini E. (2010). Effects of selected pectinolytic bacterial strains on water-retting of hemp and fibre properties. *J. Appl. Microbiol.* 108 194–203. 10.1111/j.1365-2672.2009.04409.x 19558465

[B23] Gibson Ronald F. (2016). *Principles of Composite Material Mechanics.* Boca Raton, FL: CRC press.

[B24] González-García S. Hospido A. Feijoo G. Moreira M. T. (2010). Life cycle assessment of raw materials for non-wood pulp mills: hemp and flax. *Resour. Conserv. Recycl.* 54 923–930. 10.1016/j.resconrec.2010.01.011

[B25] Grassi G. McPartland J. M. (2017). *Chemical and Morphological Phenotypes in Breeding of Cannabis sativa L. Cannabis sativa L.-Botany and Biotechnology.* Cham: Springer, 137–160. 10.1007/978-3-319-54564-6_6

[B26] Jankauskienė Z. Butkute B. Gruzdeviene E. Cesevičienė J. Fernando A. L. (2015). Chemical composition and physical properties of dew- and water-retted hemp fibers. *Ind. Crops Prod.* 75 206–211. 10.1016/j.indcrop.2015.06.044

[B27] Keller A. Leupin M. Mediavilla V. Wintermantel E. (2001). Influence of the growth stage of industrial hemp on chemical and physical properties of the fibres. *Ind. Crops Prod.* 13 35–48. 10.1016/S0926-6690(00)00051-0

[B28] Li Y. Pickering K. L. Farrell R. L. (2009). Analysis of green hemp fibre reinforced composites using bag retting and white rot fungal treatments. *Ind. Crops Prod.* 29 420–426. 10.1016/j.indcrop.2008.08.005

[B29] Li Y. Pickering K. L. (2009). The effect of chelator and white rot fungi treatments on long hemp fibre-reinforced composite’. *Compos. Sci. Technol.* 69 1265–1270. 10.1016/j.compscitech.2009.02.037

[B30] Linger P. Müssig J. Fischer H. Kobert J. (2002). Industrial hemp (*Cannabis sativa* L.) growing on heavy metal contaminated soil: fibre quality and phytoremediation potential. *Ind. Crops Prod.* 16 33–42. 10.1016/S0926-6690(02)00005-5

[B31] Liu M. Ale M. T. Kołaczkowski B. Fernando D. Daniel G. Meyer A. S. (2017). Comparison of traditional field retting and *Phlebia radiata* Cel 26 retting of hemp fibres for fibre-reinforced composites. *AMB Express* 7:58. 10.1186/s13568-017-0355-8 28275995 PMC5342995

[B32] Liu M. Baum A. Odermatt J. Berger J. Yu L. Zeuner B. (2016). Oxidation of lignin in hemp fibers by laccase: effects on mechanical properties of hemp fibers and unidirectional fiber/epoxy composites. *Compos. Part A Appl. Sci. Manuf.* 95 377–387. 10.1016/j.compositesa.2017.01.026

[B33] Liu M. Fernando D. Daniel G. Madsen B. Meyer A. S. Ale M. T. (2015). Effect of harvest time and field retting duration on the chemical composition, morphology and mechanical properties of hemp fibers. *Ind. Crops Prod.* 69 29–39. 10.1016/j.indcrop.2015.02.010

[B34] Madsen B. Thygesen A. Lilholt H. (2009). Plant fibre composites– porosity and stiffness. *Compos. Sci. Technol.* 69 1057–1069. 10.1016/j.compscitech.2009.01.016

[B35] Munder F. Fürll C. Hempel H. (2004). Results of an advanced technology for decortication of hemp, flax and linseed. *Mol. Cryst. Liq. Cryst.* 418 37–41. 10.1080/15421400490479253

[B36] Müssig J. (2001). *Untersuchung der Eignung heimischer Pflanzenfasern fur die Herstellung von naturfaserverstärkten’ Duroplasten: vom Anbau zum Verbundwerkstoff.* Dusseldorf: VDI Verlag.

[B37] Müssig J. Amaducci S. (2018). Scanner based image analysis to characterise the influence of agronomic factors on hemp (*Cannabis sativa* L.) fibre width. *Ind. Crops Prod.* 113 28–37. 10.1016/j.indcrop.2017.12.059

[B38] Müssig J. (ed.) (2010). *Industrial Applications of Natural Fibres.* Hoboken, NJ: Wiley 10.1002/9780470660324

[B39] Müssig J. Martens R. (2003). Quality aspects in hemp fibre production– influence of cultivation, Harvesting and Retting. *J. Ind. Hemp* 8 11–32. 10.1300/J237v08n01 15081488

[B40] Pecenka R. Furll C. Idler C. Grundmann P. Radosavljevic L. (2009). Fibre boards and composites from wet preserved hemp. *Int. J. Mater. Prod. Technol.* 36 208–220. 10.1504/IJMPT.2009.027832

[B41] Placet V. (2009). Characterization of the thermo-mechanical behaviour of Hemp fibres intended for the manufacturing of high performance composites. *Compos. Part A Appl. Sci. Manuf.* 40 1111–1118. 10.1016/j.compositesa.2009.04.031

[B42] Placet V. Day A. Beaugrand J. (2017). The influence of unintended field retting on the physicochemical and mechanical properties of industrial hemp bast fibres. *J. Mater. Sci.* 52 5759–5777. 10.1007/s10853-017-0811-5

[B43] Prade T. Svensson S. E. Mattsson J. E. (2012). Energy balances for biogas and solid biofuel production from industrial hemp. *Biomass Bioenergy* 40 36–52. 10.1016/j.biombioe.2012.01.045

[B44] R Core Team (2012). *R: A Language and Environment for Statistical Computing.* Vienna: R Foundation for Statistical Computing.

[B45] Ranalli P. Venturi G. (2004). Hemp as a raw material for industrial applications. *Euphytica* 140 1–6. 10.1007/s10681-004-4749-8 15081488

[B46] Ribeiro A. Pochart P. Day A. Mennuni S. Bono P. Baret J. L. (2015). Microbial diversity observed during hemp retting. *Appl. Microbiol. Biotechnol.* 99 4471–4484. 10.1007/s00253-014-6356-5 25575888

[B47] Saleem Z. Rennebaum H. Pudel F. Grimm E. (2008). Treating bast fibres with pectinase improves mechanical characteristics of reinforced thermoplastic composites. *Compos. Sci. Technol.* 68 471–476. 10.1016/j.compscitech.2007.06.005

[B48] Sankari H. S. (2000). Comparison of bast fibre yield and mechanical fibre properties of hemp (*Cannabis sativa* L.) cultivars. *Ind. Crops Prod.* 11 73–84. 10.1016/S0926-6690(99)00038-2

[B49] Schnegelsberg G. (1999). *Handbuch der Faser– Theorie und Systematik der Faser* Vol. 1 Frankfurt am Main: Deutscher Fachverlag.

[B50] Shahzad A. (2011). Hemp fiber and its composites–a review. *J. Compos. Mater.* 46 973–986. 10.1177/0021998311413623

[B51] Slootmaker T. Müssig J. (2010). “SEM catalogue for animal and plant fibres,” in *Industrial Applications of Natural Fibres*, ed. Müssig J. (Hoboken, NJ: John Wiley & Sons), 311–336. 10.1002/9780470660324.ch14

[B52] Tamburini E. León A. G. Perito B. Mastromei G. (2003). Characterization of bacterial pectinolytic strains involved in the water retting process. *Environ. Microbiol.* 5 730–736. 10.1046/j.1462-2920.2003.00462.x 12919408

[B53] Tang K. Struik P. C. Yin X. Calzolari D. Musio S. Thouminot C. (2017). A comprehensive study of planting density and nitrogen fertilization effect on dual-purpose hemp (*Cannabis sativa* L.) cultivation. *Ind. Crops Prod.* 107 427–438. 10.1016/j.indcrop.2017.06.033

[B54] Tang K. Struik P. C. Yin X. Thouminot C. Bjelková M. Stramkale V. (2016). Comparing hemp (*Cannabis sativa* L.) cultivars for dual-purpose production under contrasting environments. *Ind. Crops Prod.* 87 33–44. 10.1016/j.indcrop.2016.04.026

[B55] Taylor P. Karus M. Kaup M. (2005). Natural fiber composites in the European automotive industry. *J. Ind. Hemp* 7 119–131. 10.1300/J237v07n01

[B56] Thomsen A. B. Rasmussen S. Bohn V. Nielsen K. V. Thygesen A. (2005). *Hemp Raw Materials: The Effect of Cultivar, Growth Conditions and Pre-treatment on the Chemical Composition of the Fibres.* Roskilde: Risø-R Report, 6–30.

[B57] Toonen M. A. J. Maliepaard C. Reijmers T. H. Van Der Voet H. Mastebroek H. D. Van Den Broeck H. C. (2004). Predicting the chemical composition of fibre and core fraction of hemp (*Cannabis sativa* L.). *Euphytica* 140 39–45. 10.1007/s10681-004-4753-z

[B58] Turunen L. van der Werf H. M. G. (2006). Life cycle analysis of hemp textile yarn, comparison of three hemp fiber processing scenarios and a flax scenario. *J. Ind. Hemp* 12 43–66. 10.1300/J237v12n02_04

[B59] van den Broeck H. C. Maliepaard C. Ebskamp M. J. M. Toonen M. A. J. Koops A. J. (2008). Differential expression of genes involved in C1 metabolism and lignin biosynthesis in wooden core and bast tissues of fibre hemp (*Cannabis sativa* L.). *Plant Sci.* 174 205–220. 10.1016/j.plantsci.2007.11.008

[B60] Venturi P. Amaducci S. Amaducci M. T. Venturi G. (2007). Interaction between agronomic and mechanical factors for fiber crops harvesting: Italian results. *Note II. Hemp J. Nat. Fibers* 4 83–97. 10.1300/J395v04n03_06

[B61] Wang S. Gusovius H. J. Lühr C. Musio S. Uhrlaub B. Amaducci S. (2018). Assessment system to characterise and compare different hemp varieties based on a developed lab-scaled decortication system. *Ind. Crops Prod.* 117 159–168. 10.1016/j.indcrop.2018.02.083

[B62] Westerhuis W. Amaducci S. Struik P. C. Zatta A. Van Dam J. E. G. Stomph T. J. (2009). Sowing density and harvest time affect fibre content in hemp (*Cannabis sativa*) through their effects on stem weight. *Ann. Appl. Biol.* 155 225–244. 10.1111/j.1744-7348.2009.00334.x

[B63] Wötzel K. Wirth R. Flake M. (1999). Life cycle studies on hemp fibre reinforced components and ABS for automotive parts. *Die Angewandte Makromolekulare Chemie* 272 121–127. 10.1002/(SICI)1522-9505(19991201)272:1<121::AID-APMC121>3.0.CO;2-T

[B64] Yan Z. L. Wang H. Lau K. T. Pather S. Zhang J. C. Lin G. (2013). Reinforcement of polypropylene with hemp fibres. *Compos. Part B Eng.* 46 221–226. 10.1016/j.compositesb.2012.09.027

[B65] Zatta A. Monti A. Venturi G. (2012). Eighty years of studies on industrial hemp in the Po Valley (1930-2010). *J. Nat. Fibers* 9 180–196. 10.1080/15440478.2012.706439

